# Analyses of circRNA profiling during the development from pre-receptive to receptive phases in the goat endometrium

**DOI:** 10.1186/s40104-019-0339-4

**Published:** 2019-04-25

**Authors:** Yuxuan Song, Lei Zhang, Xiaorui Liu, Mengxiao Niu, Jiuzeng Cui, Sicheng Che, Yuexia Liu, Xiaopeng An, Binyun Cao

**Affiliations:** College of Animal Science and Technology, Northwest A&F University, Yangling, Shaanxi 712100 People’s Republic of China

**Keywords:** CircRNAs, Dairy goat, Endometrial epithelium cells, Endometrial stromal cells, Receptive endometrium

## Abstract

**Background:**

Recent studies have revealed that noncoding RNAs play important regulatory roles in the formation of endometrial receptivity. Circular RNAs (circRNAs) are a universally expressed noncoding RNA species that have been recently proposed to act as miRNA sponges that directly regulate expression of target genes or parental genes.

**Results:**

We used Illumina Solexa technology to analyze the expression profiles of circRNAs in the endometrium from three goats at gestational day 5 (pre-receptive endometrium, PE) and three goats at gestational day 15 (receptive endometrium, RE). Overall, 21,813 circRNAs were identified, of which 5,925 circRNAs were specific to the RE and 9,078 were specific to the PE, which suggested high stage-specificity. Further analysis found 334 differentially expressed circRNAs in the RE compared with PE (*P* < 0.05). The analysis of the circRNA-miRNA interaction network further supported the idea that circRNAs act as miRNA sponges to regulate gene expression. Moreover, some circRNAs were regulated by estrogen (E2)/progesterone (P4) in endometrial epithelium cell lines (EECs) and endometrial stromal cell line (ESCs), and each circRNA molecule exhibited unique regulation characteristics with respect to E2 and P4.

**Conclusions:**

These data provide an endometrium circRNA expression atlas corresponding to the biology of the goat receptive endometrium during embryo implantation.

**Electronic supplementary material:**

The online version of this article (10.1186/s40104-019-0339-4) contains supplementary material, which is available to authorized users.

## Background

In mammals, endometrial receptivity is essential for successful embryo implantation [1]. The receptive endometrium (RE) is a spatial and temporal phenomenon known as the “window of implantation” [2, 3] that is an absolutely necessary part of the reproductive process. During the “window of implantation”, the endometrium undergoes remarkable changes in construction and function: endometrial stromal cells (ESCs) proliferate, endometrial epithelium cells (EECs) differentiate, and the endometrium acquires adhesive properties that enable embryo adhesion and subsequent invasion [4–6]. Many researchers have proposed that the failure of *in vitro* fertilization embryo transfer using good quality embryos is due to impaired RE [7, 8] or inappropriate epigenetic modifications [9]. Therefore, more studies are urgently needed to explore the molecular regulation mechanisms of RE to improve the success rate of embryo implantation and the fecundity of females, which is one of the most economically important components in animal husbandry [10, 11].

The number of genes and proteins identified from previous studies that are involved in the establishment of RE has exponentially increased [12, 13]. The establishment of RE is a highly dynamic process that is post-transcriptionally regulated, and several epigenetically regulated genes in the endometrium have been identified [14]. Recent studies have demonstrated that noncoding RNAs play important roles in the formation of RE, and we have previously identified 143 differentially expressed miRNAs in the RE compared to the PE [15]. Moreover, another study demonstrated that hsa-miR-30b, hsa-miR-30d, and hsa-miR-494 play important roles in gene reprogramming during the formation of RE in humans [5].

With potential functions as competing endogenous RNAs or miRNA sponges, circRNAs contain miRNA response elements (MREs) that enable them to act as molecular sponges for miRNAs leading to the derepression of miRNA target genes, thereby influencing post-transcriptional regulation [16]. The circRNAs from back-spliced exons have been identified as a naturally occurring family of noncoding RNAs in eukaryotes [17, 18]. In recent years, circRNAs have been the focus of international research, and a large number of circRNAs have been successfully identified in various cell lines and species [19–22]. Furthermore, studies have demonstrated that circRNAs are expressed in eutopic and normal endometrium [23], endometriosis [24], and endometrial cancer [25]. However, there is currently no information available on the expression and function of circRNAs in the developing normal endometrium, or on the differences in circRNAs expression between the RE and PE.

Considering the universal expression of circRNAs and their key role in regulating gene expression, we hypothesized that circRNAs are expressed in the endometrium of dairy goats as potential regulators of RE at the post-transcriptional level. Focusing on these points, we investigated the circRNA expression profile in the endometrium at gestational day 5 (PE) and gestational day 15 (RE) using Illumina Solexa technology. In addition, we analyzed the differentially expressed circRNAs (DECs) in the RE and PE and conducted GO enrichment and KEGG pathway analyses of the hosting genes of DECs (hg-DECs). Then, we examined the regulatory relationships between circRNAs with miRNAs and corresponding mRNAs.

## Methods

### Ethics statement and sample collection

All experimental goats were provided by XinLongMen milk goat breeding Farm, Xi’an, Shaanxi Province, China. The goats were maintained according to the No. 5 Proclamation of the Ministry of Agriculture, P. R. China, and we confirm that all animal protocols and methods were approved by the Review Committee for the Use of Animal Subjects of Northwest A&F University. A total of 10 healthy, 24-month-old multiparous dairy goats (Xinong Saanen) were treated as be described previously [15, 26]. Endometrium samples from 2 goats at gestational day 5 (pre-receptive endometrium, PE) and 2 goats at gestational day 15 (receptive endometrium, RE) were obtained from the anterior wall of the uterine cavity. What’s more, the other 6 goats were treated to verify the results of RNA-Seq.

### RNA-Seq analysis

Total RNA was isolated and purified using Trizol reagent (Invitrogen, Carlsbad, CA, USA) following the manufacturer’s procedure. The RNA amount and purity of each sample was quantified using NanoDrop ND-1000 (NanoDrop, Wilmington, DE, USA). The RNA integrity was assessed by Agilent 2100 with RIN number > 7.0. Approximately 5 μg of total RNA was used to deplete ribosomal RNA according to the manuscript of the Ribo-Zero™ rRNA Removal Kit (Illumina, San Diego, USA). After removing ribosomal RNAs, the remaining RNAs were fragmented into small pieces using divalent cations under 94 °C. Then the cleaved RNA fragments were reverse-transcribed to create the cDNA, which were next used to synthesise U-labeled second-stranded DNAs with *E. coli* DNA polymerase I, RNase H and dUTP. An A-base was then added to the blunt ends of each strand, preparing them for ligation to the indexed adapters. Each adapter contained a T-base overhang for ligating the adapter to the A-tailed fragmented DNA. Single-or dual-index adapters were ligated to the fragments, and size selection was performed with AMPureXP beads. After the heat-labile UDG enzyme treatment of the U-labeled second-stranded DNAs, the ligated products were amplified with PCR by the following conditions: initial denaturation at 95 °C for 3 min; 8 cycles of denaturation at 98 °C for 15 s, annealing at 60 °C for 15 s, and extension at 72 °C for 30 s; and then final extension at 72 °C for 5 min. The average insert size for the final cDNA library was 300 bp (±50 bp). At last, we performed the paired-end sequencing on an Illumina Hiseq 4000 (LC Bio, China) following the vendor’s recommended protocol. The RNA-Seq data was deposited in GEO (GSE85384).

### Sequence and primary analysis

Prior to reference genome mapping, the raw data (raw reads) were processed to remove low quality reads. In this step, clean reads (valid data) were obtained by removing reads containing adapter sequences, the uncertain base number in a read was more than 5%, low quality reads (1, reads containing sequencing adaptors; 2, reads containing sequencing primer; 3, nucleotide with *q*-value < 20). All of the downstream analyses were based on high quality clean data.

### RNA-Seq reads mapping

CircRNAs are thought to be predominantly produced by back-splicing reactions that covalently link the 3′ splice donor (3′ end) of an downstream exon to the 5′ splice acceptor (5′ end) of an upstream exon [27, 28]. Due to the rearranged exon ordering, specific algorithms are required to annotate these back-spliced exon events for circular RNA prediction. Firstly, the clean reads (valid data) were mapped to the goat reference genome (http:/www.gencodegenes.org/, GRCh38) using a publicly available program TopHat 2 (http://ccb.jhu.edu/software/tophat/index.shtml) [29], what aligns the reads to genomes using the ultra high-throughput short read aligner Bowtie2 [30], and then analyzes the mapping results to identify splice junctions between exons.

Secondly, the unmapped reads with TopHat but could be mapped with TopHat-Fusion (http://ccb.jhu.edu/software/tophat/fusion_index.shtml) on the same chromosome in a noncolinear ordering (back-spliced ordering) were extracted as candidate back-spliced junction reads. Last, the circRNAs were identified from back-splice junction reads based on structural and splice sites characters of circRNAs: (1) the two ends of splice sites must be GU/AG, (2) mismatch≤2, (3) back-splice junction reads ≥1, (4) if there were more than 2 splice sites, they were on the same chromosome and not too wide apart from each other more than 100 kb.

### Identification and quantification of goat circRNAs

Cufflinks software was used to assemble circRNAs, estimate their abundances, and test for differential expression, and the unit of the measurement is Fragment Per Kilobase of exon per Million fragments mapped (FPKM) [31] in this study. Only the comparisons with “*q*-value” less than 0.01 and status marked as “OK” in the Cuffdiff output were regarded as showing differential expression.

### GO enrichment and KEGG pathway analysis of circRNA-hosting gene

The hypergeometric test was applied to map all differentially expressed genes to terms in the Gene Ontology (GO) database (ftp://ftp.ncbi.nih.gov/gene/DATA/gene2go.gz) [32], is an international standard gene functional classification system [33]. The corrected *P*-value< 0.05 was used as the threshold to find significantly enriched GO terms in the input list of hgDECs compared to their genomic background [26]. In addition, GO DAG, what illustrated the relationships of the GO terms [34], were explained the using cytoscape 2.8.1 version [35].

KEGG helped researchers to better understanding the biological functions of genes based on large-scale molecular datasets (http://www.genome.jp/kegg/) [36]. In this study, the corrected *P*-value< 0.05 was used as a threshold [37] to find significantly enriched KEGG terms in the input list of DEGs compared to their genomic background [38].

### The interaction network analysis of circRNA-miRNA

CircRNAs are believed to contribute substantially to the competing endogenous RNAs network for the studies what reported it acted as miRNA sponges to regulate gene expression [16, 39]. In this study, two publicly available program, Targetscan 7.0 (http://www.targetscan.org/vert_72/ and miRanda (http://www.microrna.org/microrna/home.do), were used to predict the interaction of circRNA-miRNA. The potential regulated relationship between the DECs in the present study and the goat miRNAs were also predicted by Targetscan 7.0 and miRanda.

### RNA preparation and RT-qPCR

After sequencing, the other 6 goats (3 goats at day 5, and 3 goats at day 15) were treated to verify the results of RNA-Seq using RT-qPCR. Total RNA from the goat endometrium were extracted using Trizol reagent (TaKaRa, Dalian, China), 1% agarose gel electrophoresis and spectrometry (A260/A280) ratio were detected to judge the RNA quality [40–42]. And then the RNA was incubated for 15 min at 37 °C with or without (mock) 3 U/μg of RNase R (Epicenter Biotechnologies, Chicago, USA). To quantify the amount of circRNA, cDNA was synthesized with the Prime Script RT reagent Kit with gDNA Eraser (TaKaRa, Dalian, China) using random hexamers. In particular, the divergent primers annealing at the distal ends of circRNA and RT-qPCR were used to determine the abundance of circRNA. GAPDH was used as the reference, the relative expression levels of the mRNAs were calculated using the eq. *N* = 2^-ΔΔCt^. What’s more, the PCR products from the individual circRNAs were further identified by Sanger sequencing.

### Cell culture and treatment

Caprine endometrial epithelial cell (EEC) and stromal cell (ESC) lines were kindly provided by Dr. Y.P. Jin (Northwest A&F University, Yangling, China) [43]. Cells were cultured and maintained in Dulbecco′s Modified Eagle Medium (DMEM)-Hank F12 (Gibco, Invitrogen Corporation, Grand Island, NY, USA) supplemented with 10% charcoal stripped fetal bovine serum (FBS; Gibco, USA) and antibiotics (100 U/mL penicillin and 100 mg/mL streptomycin) at 37 °C in a 5% CO_2_ atmosphere [44]. The cells were treated with different concentrations of estradiol (E2, Selleck, Catalog No. S1709) and progesterone (P4, Selleck, Catalog No. S1705) respectively (Additional file 1: Table S1), total RNAs were extracted from cells at 24 h. And then, the expression levels of some circRNAs were detected as mentioned above.

## Results

### Overview of sequencing data

To systematically identify circRNAs and their changes in expression levels between the PE and RE in Xinong Saanen dairy goats, we purified and sequenced RNA using the Illumina paired-end RNA-Seq approach. First, we isolated total RNA from 3 PE tissues and 3 RE tissues, then we ribosomal RNA-depleted and RNase R-treated the total RNA, generating a total of 120 million paired-end reads of 250 (± 50) bp in length. This approach yielded 18 Gb of sequence, representing approximately seven times the size of the genome (2.66 Gb). The distribution and uniformity of the repeated samples were analyzed using principal component analysis (Additional file 2: Figure S1A), and a boxplot was used to identify outliers and evaluate the overall quality (Additional file 2: Figure S1B). The box-plot distribution of the log_10_ FPKM (Fragment Per Kilobase of exon per Million fragments mapped) values suggested that the median and quartile values among the samples compared in terms of differential expression were almost identical. Thus, these two samples were discarded for poor repeatability, and ~ 90 million clean reads (~14Gb) were obtained after the low-quality data were separated out and discarded. A detailed summary for each sample is provided in Table 1.

**Table 1:** The overview of the results of RNA-Seq in each sample

Sample	Raw data		Valid data		Valid ratio, %	Q20, %	Q30, %	GC content, %
	Read	Base	Read	Base				
PE1	120,000,000	18.00G	97,237,044	14.59G	81.03	99.48	94.62	49
PE2	120,000,000	18.00G	90,947,840	13.64G	75.79	99.44	94.36	52.5
RE1	120,000,000	18.00G	93,492,408	14.02G	77.91	99.33	93.68	50
RE2	120,000,000	18.00G	94,273,656	14.14G	78.56	99.41	93.82	51

### The reference genome mapping

**Fig. 1:**
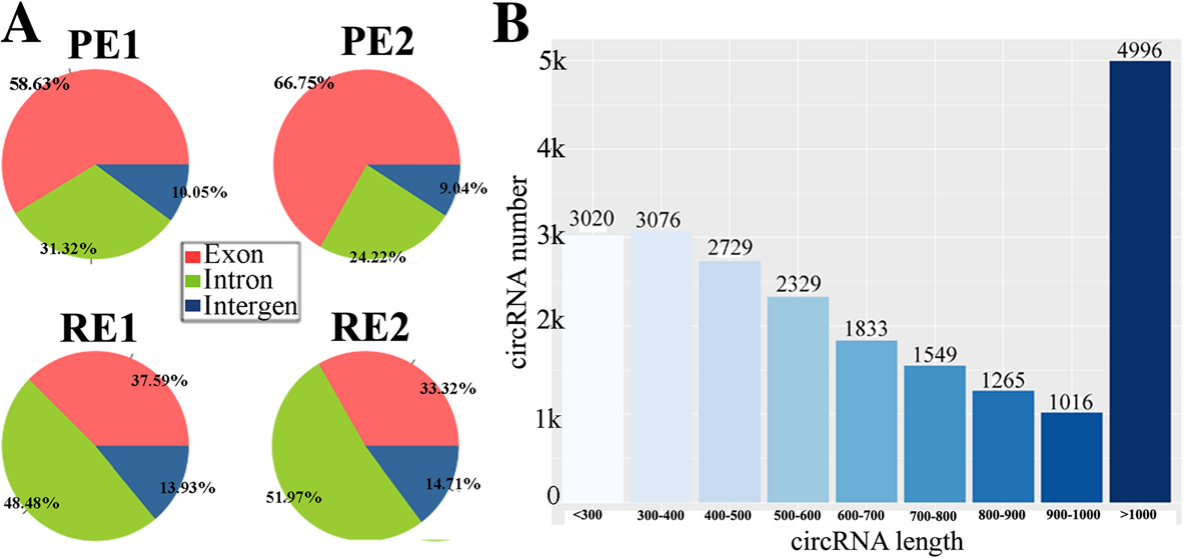
Genome and length distribution of circRNAs. Note: **a** showed the genome distribution of circRNAs, ~ 60% of the circRNAs in PE was annotated to exon and the percentages felt to ~ 30% in RE. **b** was the length distribution of circRNAs, and 4996 circRNAs (23%) were more than 1 kb in length

The clean reads (valid data, Table 1) of every sample were aligned to the goat reference genome. TopHat-Fusion was used for the nonlinear alignment of the back-splice junction reads (Additional file 3). Moreover, circRNAs can arise from exons or introns, and these distinct species show independent modes of generation. Thus, the reads mapped to the genome were divided into three groups (exonic, intronic, and intergenic) based on their origin (Fig. 1a). Furthermore, approximately 23% circRNAs were more than 1 kb in length (Fig. 1b).

### Identification of goat circRNAs

In this study, the circRNA number and the corresponding circRNA-hosting gene number in every sample are shown in Table 2, and a total of 21,813 circRNAs were identified (Additional file 4). More than 95% of the circRNAs (20,720 circRNAs) consisted of protein coding exons, and only 5% of the circRNAs (1,093) identified were circular intronic RNAs (ciRNAs). We annotated these circRNA candidates based on the location of the corresponding circRNA-hosting genes, and 5,457 corresponding circRNA-hosting genes were mapped to the goat genome.

**Table 2:** Number of circRNAs and circRNA-hosting genes in goat endometrium

Sample	PE1	PE2	RE1	RE2	Total
Candidate back-spliced junction reads^a^	924,831 (0.95%)	911,567 (1.00%)	1,490,765 (1.59%)	1,895,346 (2.01%)	/
Condident post reads^b^	30,793 (0.03%)	70,062 (0.08%)	31,717 (0.03%)	44,567 (0.05%)	/
CircRNA number^c^	7,968	12,114	6,980	9,188	21,813
CircRNA-hosting gene number^d^	3,369	4,160	3,123	3,563	5,457

^a^ the number of candidate back-spliced junction reads; ^b^ the number of the A after the reads were adjusted and filtered based on the sequence features of back-spliced junction; ^c^ the number of circRNAs; ^d^ the number of circRNA-hosting genes

### Differential expression of circRNAs in the receptive and pre-receptive endometrium

The expression levels of circRNAs were normalized by FPKM value, and the FPKM distribution of circRNA in each sample is shown in Table 3. More than 80% of the circRNAs were detected at medium expression levels (FPKM), and approximately 2% of the circRNAs were found to be highly expressed in the PE and RE libraries, respectively.

**Table 3:** The interval analysis of circRNA expression (FPKM)

	Sample	PE1	PE2	RE1	RE2
Lowly expressed circRNAs^a^	0–0.1 FI	20 (0.25%)	58 (0.48%)	18 (0.26%)	35 (0.38%)
	0.1–0.3 FI	84 (1.05%)	147 (1.21%)	51 (0.73%)	89 (0.97%)
Medium expressed circRNAs^b^	0.3–3.57 FI	2,523 (31.66%)	4,506 (37.20%)	3,284 (47.05%)	3,116 (33.91%)
	3.57–15 FI	3,861 (48.46%)	5,663 (46.75%)	2,995 (42.91%)	4,504 (49.02%)
Highly expressed circRNAs^c^	15–60 FI	1,314 (16.49%)	1,523 (12.57%)	563 (8.07%)	1,288 (14.02%)
	> 60 FI	166 (2.08%)	217 (1.79%)	69 (0.99%)	156 (1.70%)

FPKM value (Fragment Per Kilobase of exon per Million fragments mapped) were categorized into six groups in this study. *FI* FPKM interval. ^a^ circRNAs expressional level < 0.3 RPKM; ^b^ circRNAs expressional level ranging from 0.3 to 15 RPKM. ^c^ circRNAs expressional level > 15 RPKM

**Fig. 2:**
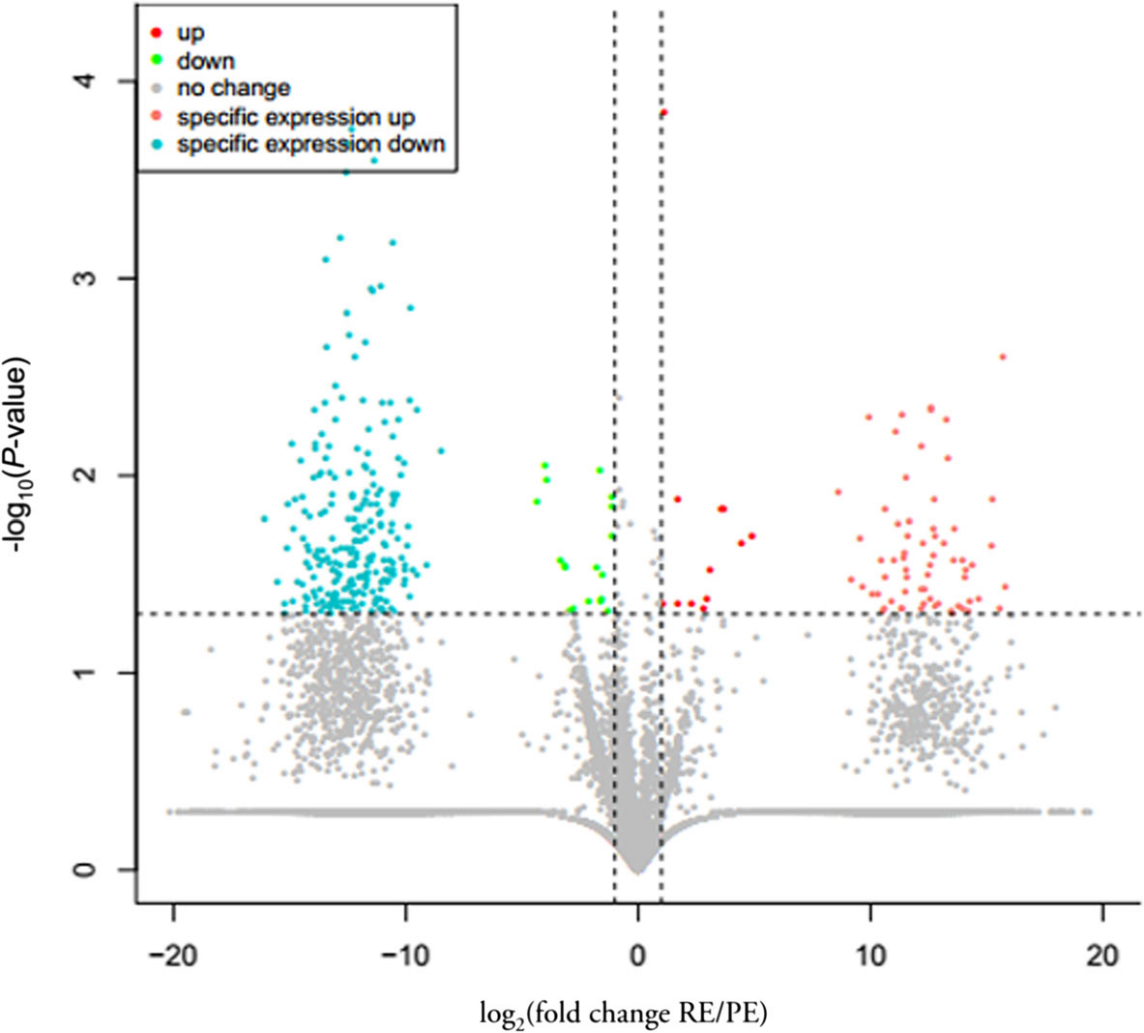
The detected circRNAs in PE and RE of dairy goat. Note: Volcano plot showing *P* values (−log_10_) versus circRNAs ratio of RE/PE (log_2_). Red, 12 up-regulated circRNAs; green, 18 down-regulated circRNAs; gray, 21,479 not significantly changed circRNAs; pink, 65 specifically in RE; blue, 239 specifically in PE. Fisher’s exact test *P* < 0.05

The fold changes (log_2_(PE/RE)) and corresponding significance threshold of the *P*-value were estimated according to the normalized circRNAs expression levels. Based on the expression levels, the DECs between the PE and RE were considered significant when *P* < 0.05. A total of 334 circRNAs significantly differed in terms of FPKM levels between the PE and RE (Additional file 5). Overall, 257 differentially expressed circRNAs were downregulated, whereas 77 circRNAs were upregulated in the RE compared to the PE. Further analysis showed that 30 of the 334 DECs were co-expressed in the two physiological stages of the goat endometrium, whereas 65 DECs were specifically expressed in the RE and 239 were specifically expressed in the PE (Fig. 2, Additional file 6).

**Fig. 3:**
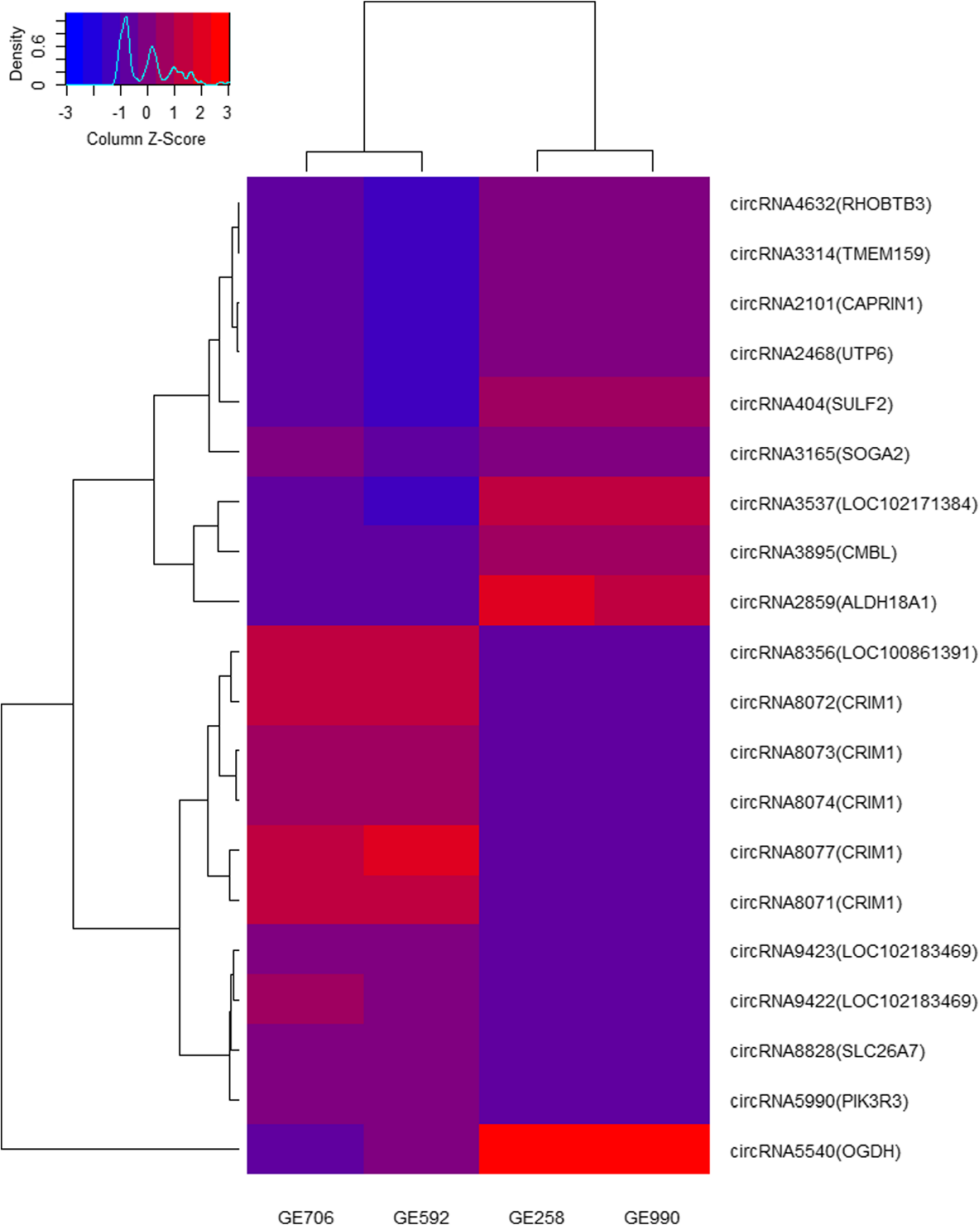
Clustering analysis of differentially expressed circRNAs with 10 higher expression levels. Note: Heat map of Pearsons correlation across the differentially expressed circRNAs with top 10 highest FPKM value in RE and top 10 in PE, and their corresponding hosting genes are showed in the parenthesis. A dendrogram of correlation between circRNAs is shown to the left of the heatmap

The ten most upregulated circRNAs in RE compared to PE with the ten highest FPKM values are shown in Table 4. The FPKM value of circRNA8077 was highest (FPKM value = 57.95) in the RE, followed by circRNA8071 (FPKM value = 53.65). Notably, five of the ten upregulated circRNAs in the RE were derived from cysteine-rich motor neuron 1 (*CRIM1*), revealing that one gene could produce multiple circRNAs. The downregulated circRNAs with the ten highest FPKM values in the PE are shown in Table 5. The highest was circRNA5540 (FPKM value = 138.64, 15.37-fold decrease) in the PE, followed by circRNA2859 (FPKM value = 86.99, 20.27-fold decrease), and circRNA3537 (FPKM value = 69.31). A heatmap of the Pearson’s correlation values and a dendrogram of the correlation between the 20 DECs are provided in Fig. 3.

**Table 4:** The up-regulated circRNAs with top 10 highest FPKM value in RE

Accession No.	PE1	PE2	RE1	RE2	Mean RE	Mean PE	log_2_ (fold change)	*P*-value	Isoform name	Gene name
circRNA5990	6.7	2.88	21	26	23.56	4.78	2.3014 (4.93)	0.04502	XM_005678520.1	*PIK3R3*
circRNA8071	0	0	54	53	53.65	0	Inf	0.00245	XM_005686534.1	*CRIM1*
circRNA8072	0	3.06	47	47	46.79	1.53	4.93635 (30.62)	0.02029	XM_005686534.1	*CRIM1*
circRNA8073	0	0	41	39	39.82	0	Inf	0.01318	XM_005686534.1	*CRIM1*
circRNA8074	0	0	40	37	38.61	0	Inf	0.02234	XM_005686534.1	*CRIM1*
circRNA8077	0	0	61	55	57.95	0	Inf	0.03666	XM_005686534.1	*CRIM1*
circRNA8356	0	0	45	53	49.11	0	Inf	0.04748	XM_005685948.1	*LOC100861391*
circRNA8828	0	0	20	22	21.44	0	Inf	0.02856	XM_005689247.1	*SLC26A7*
circRNA9422	0	0	25	28	26.31	0	Inf	0.04159	XM_005696368.1	*LOC102183469*
circRNA9423	0	3.75	23	27	24.73	1.87	3.72163 (13.19)	0.01463	XM_005696368.1	*LOC102183469*

*Inf* mean that the circRNA was specifically expressed in RE

**Table 5:** The down-regulated circRNAs with top 10 highest FPKM value in PE

Accession No.	PE1	PE2	RE1	RE2	Mean RE	Mean PE	log_2_ (fold change)	*P*-value	Isoform name	Gene name
circRNA2101	36	33.8	0	0	0	35.13	-Inf	0.02343	XM_005690075.1	*CAPRIN1*
circRNA2468	36	34.1	0	0	0	34.81	-Inf	0.01383	XM_005693233.1	*UTP6*
circRNA2859	93	80.6	8.6	0	4.29	86.99	−4.3413 (20.27)	0.0136	XM_005698259.1	*ALDH18A1*
circRNA3165	36	39.3	12	18	15.28	37.64	−1.3007 (2.46)	0.0479	XM_005697224.1	*SOGA2*
circRNA3314	41	35.4	0	0	0	38.03	-Inf	0.04388	XM_005697586.1	*TMEM159*
circRNA3537	68	71.1	0	0	0	69.31	-Inf	0.01638	XM_005696014.1	*LOC102171384*
circRNA3895	56	68.9	14	0	6.88	62.32	−3.179 (9.06)	0.02825	XM_005694861.1	*CMBL*
circRNA404	50	44.4	0	0	0	47.00	-Inf	0.03469	XM_005688727.1	*SULF2*
circRNA4632	41	35.2	0	0	0	38.13	-Inf	0.04937	XM_005683482.1	*RHOBTB3*
circRNA5540	129	148	18	0	9.02	138.64	−3.9416 (15.37)	0.01064	XM_005679323.1	*OGDH*

*Inf* mean that the circRNA was specifically expressed in PE

### GO and KEGG analysis of the circRNA-hosting genes

In this study, 21,813 circRNA were annotated to 5,457 circRNA-hosting genes, suggesting that a single gene might produce more than one circRNA. For example, in the goat endometrium, *CRIM1* produced twenty circRNAs, *PIK3R3* produced five circRNAs, *OGDH* produced seven circRNAs, and *ALDH18A1* produced three circRNAs (Additional file 4). Considering that a given circRNA is spliced from a given linear transcript, it is likely that the function of the circRNA is related to the function of its hosting gene [22]. Therefore, the circRNA-hosting genes were analyzed by running queries for each gene against the GO database, which provided information related to three ontologies: molecular function, cellular component, and biological process.

**Fig. 4:**
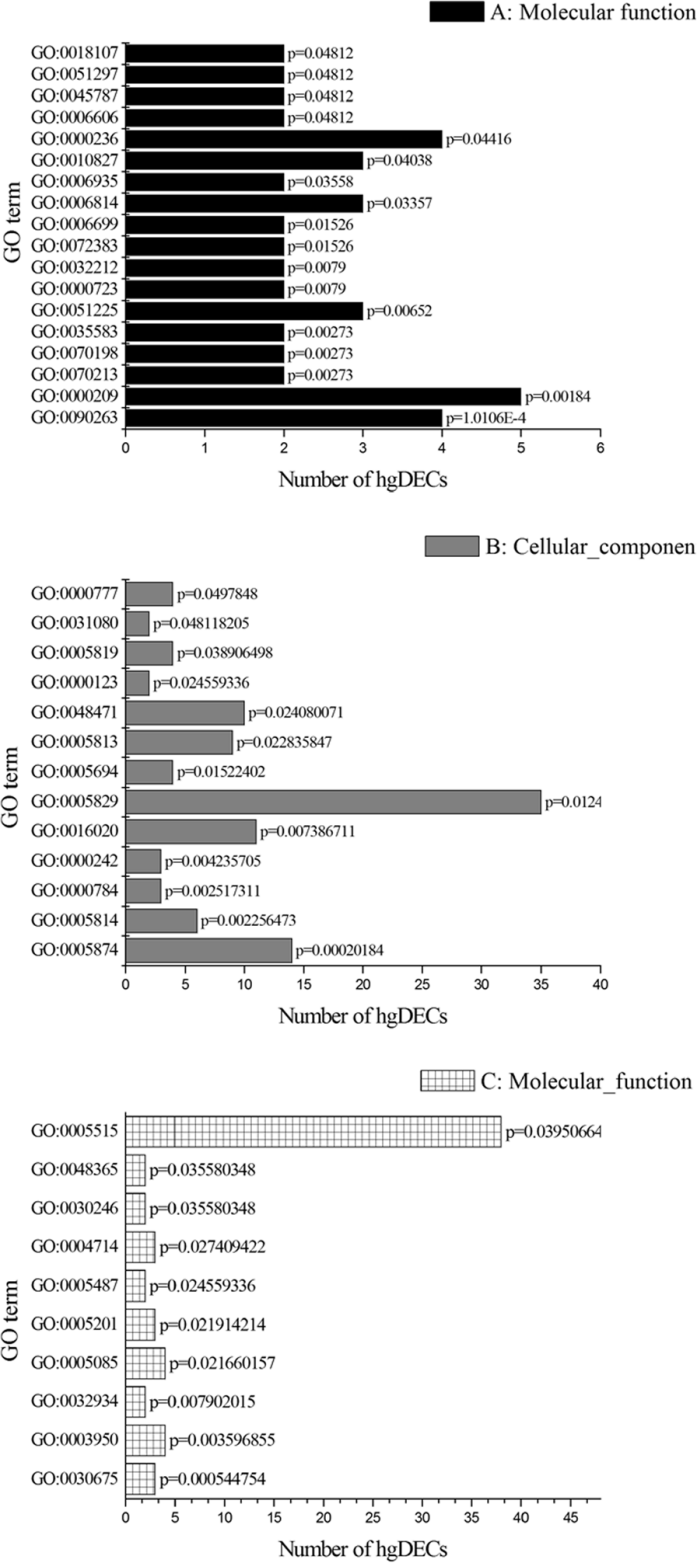
The GO enrichment analysis of predicted hosting genes of differentially expressed circRNAs with *P* < 0.05. Note: The right x-axis indicates the number of hgDECs in a category. The left y-axis indicates the specific category of GO**.** A: Biological process, B: Molecular function, C: Cellular component

Notably, 243 host genes of the 344 DECs (hg-DECs) were annotated to 850 GO terms (Additional file 7). We focused our attention on the GO terms with *P* < 0.05 and found that the hg-DECs were categorized into 41 significant GO terms (Additional file 8). Out of the 18 terms that were significantly enriched in molecular functions (Fig. 4a), the most significantly enriched GO terms were positive regulation of canonical *Wnt* receptor signaling pathway (GO:0090263) with 4 hg-DECs annotated, followed by protein polyubiquitination (GO:0000209), and protein auto-ADP-ribosylation (GO:0070213). In the cellular compartment GO category, thirteen terms were significantly enriched (Fig. 4b). The most significantly enriched GO terms were microtubule (GO:0005874) with thirteen hg-DECs annotated, followed by centriole (GO:0005814), nuclear chromosome, telomeric region (GO:0000784), and hemidesmosome (GO:0030056). As for the biological processes, ten GO terms were significantly enriched and were related to various processes (Fig. 4c) such as transcription corepressor activity (GO:0003714), Rac GTPase binding (GO:0048365), protein binding (GO:0005515), and Rac GTPase activator activity (GO:0030675).

Moreover, extracellular matrix structural constituent (GO:0005201) and integrin-mediated signaling pathway (GO:0007229) were interesting because previous studies have proposed that integrins are morphological and biochemical biomarkers of endometrial receptivity that participate in a series of important processes in extracellular matrix (ECM) remodeling in the endometrium [26]. The inclusion of the annotation for phosphatidylinositol-3,4,5-trisphosphate binding (GO:0005547) also interested us because the PI3K/AKT signaling pathway plays important roles in the development of the RE in mice and humans.

**Fig. 5:**
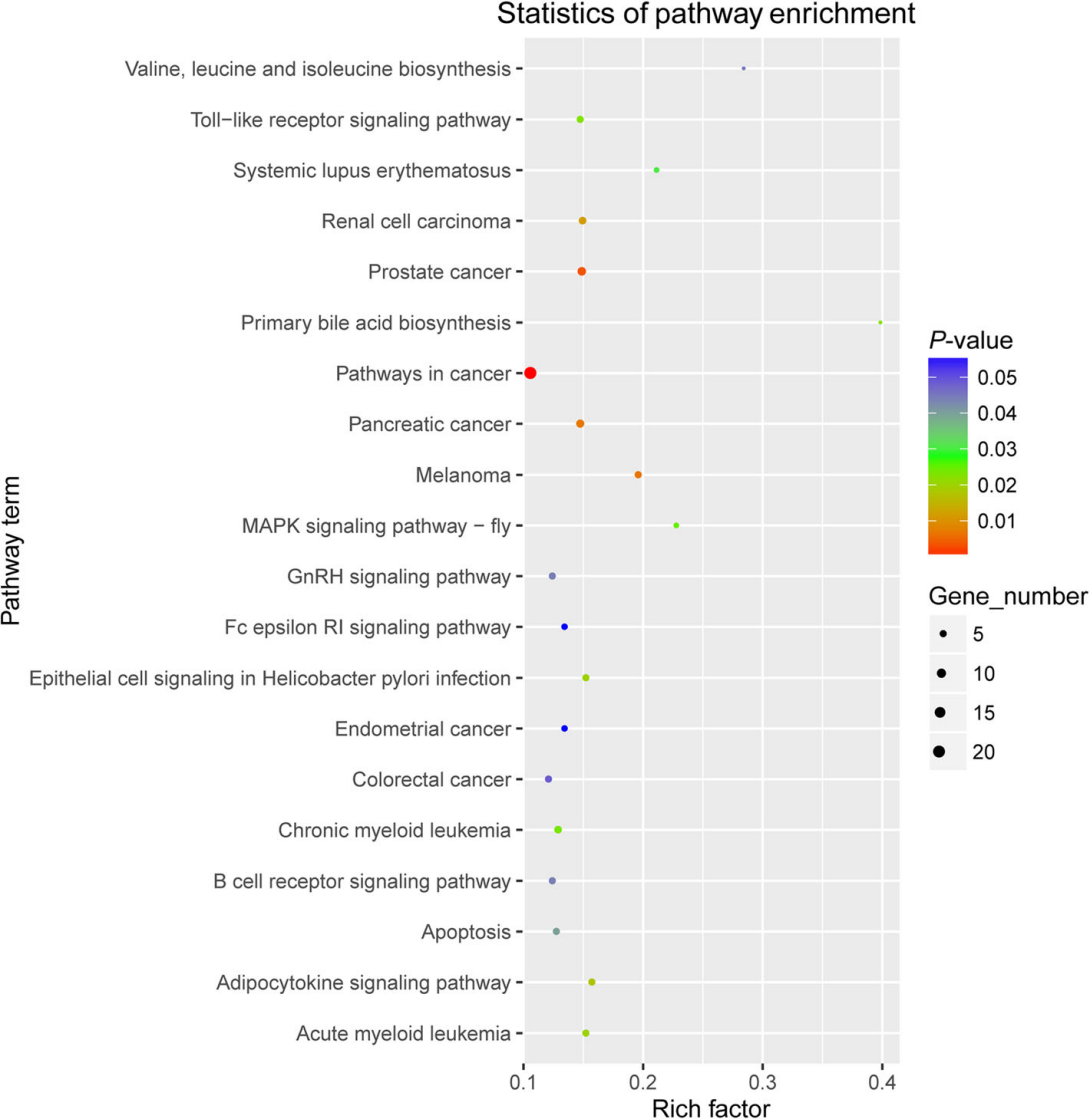
Scatter plot of the KEGG pathway enrichment analysis of hgDECs with *P* < 0.05. The x-axis indicates the number of unique sequences assigned to a specific pathway, the *y*-axis indicates the KEGG pathway. The pathway enrichment statistics were performed by Fisher’s exact test, and with a corrected *P <* 0.05 were considered the significant pathways

Various genes cooperate with each other to carry out biological functions [38]. Accordingly, KEGG analysis of circRNA-hosting genes was conducted to further understand the biological functions of circRNAs. In this study, KEGG pathway annotation showed 2,378 circRNA-hosting genes annotated to 133 biological processes (Fig. 5, Additional file 9). Moreover, the 120 hg-DECs were significantly enriched in 18 KEGG pathways (*P* < 0.05), suggesting that these pathways may play important roles in the development of endometrial receptivity (Table 6). The results showed the most significant pathways were in cancer (ko05200) with 21 hg-DECs enriched, followed by prostate cancer (ko05215), melanoma (ko05218), pancreatic cancer (ko05212), and renal cell carcinoma (ko05211). These results indicated that cancer-related pathways were active in the development of a RE from the pre-receptive phase.

**Table 6:** The 19 KEGG functional annotations for the hgDECs

Pathway ID	Pathway name	S^a^	TS^b^	*P*-value
ko05200	Pathways in cancer	21	120	0.000248705
ko05215	Prostate cancer	8	120	0.003856669
ko05218	Melanoma	5	120	0.007087531
ko05212	Pancreatic cancer	7	120	0.00724314
ko05211	Renal cell carcinoma	6	120	0.012084687
ko04920	Adipocytokine signaling pathway	5	120	0.017798454
ko05221	Acute myeloid leukemia	5	120	0.020264837
ko05120	Epithelial cell signaling in Helicobacter pylori infection	5	120	0.020264837
ko00120	Primary bile acid biosynthesis	2	120	0.022845334
ko04620	Toll-like receptor signaling pathway	5	120	0.022945388
ko05220	Chronic myeloid leukemia	6	120	0.023588462
ko04013	MAPK signaling pathway – fly	3	120	0.024723381
ko05322	Systemic lupus erythematosus	3	120	0.030330738
ko04210	Apoptosis	5	120	0.039746874
ko04662	B cell receptor signaling pathway	5	120	0.043814843
ko04912	GnRH signaling pathway	5	120	0.043814843
ko00290	Valine, leucine and isoleucine biosynthesis	2	120	0.044894146
ko05210	Colorectal cancer	5	120	0.048124857

^a^ the number of particular GO annotated hgDECs; ^b^ the number of hgDECs

### Target miRNA predictions for differentially expressed circRNAs

**Fig. 6:**
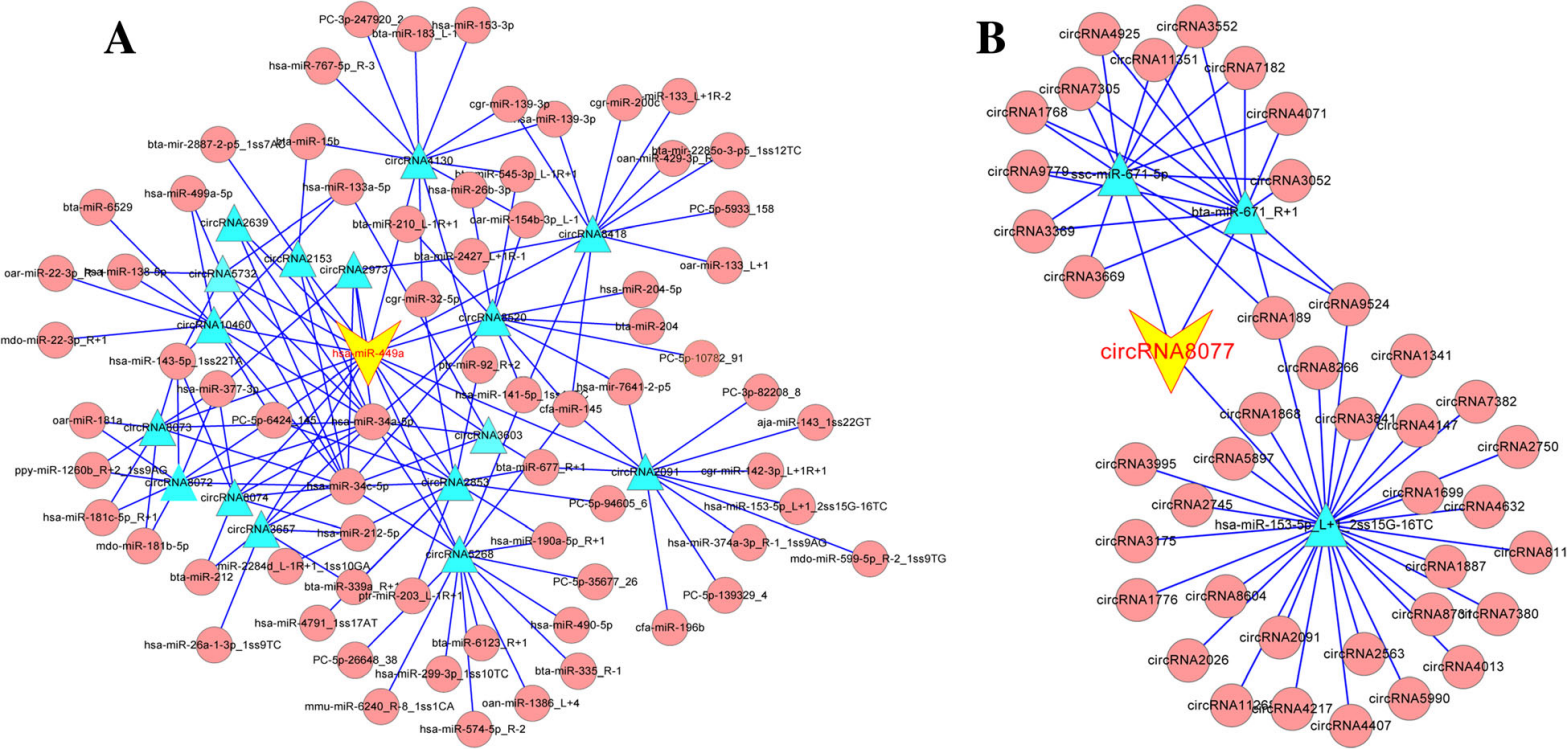
The targeting regulatory network of miRNA-circRNA centered on miRNA-449a and circRNA8077. Note: the yellow double triangle in middle place was the miRNA-449a (**a**) and circRNA8077 (**b**), blue triangle nodes represent circRNAs what were the targets predicted by two publicly available programs (Targetscan 7.0 and miRanda), and red nodes represent miRNAs what were found differentially expressed in PE and RE. The more edges a circRNA has, the more miRNAs that interact with it, and the more central a role it had within the network

There is evidence of functionality based on the finding that some exonic circRNAs act as miRNA sponges that regulate the expression of linear protein-encoding RNA products via “mRNA trap” mechanisms [17]. Two publicly available programs (Targetscan 7.0 and miRanda) were used to predict the circRNA-miRNA interactions in this study. Overall, 21,683 circRNAs were predicted as putative targets for the 436 goat miRNAs using miRBase, suggesting that a single miRNA might be regulated by more than 49 circRNAs (Additional file 10). Moreover, we observed that 308 DECs were predicted as targets of 141 differentially expressed miRNAs between the RE and PE [15]. The competing endogenous RNA (ceRNA) networks were visualized by importing the above interactions into the Cytoscape software (Fig. 6**)**. For example, miR-449a shared MREs with 16 circRNAs (Fig. 6a) and three miRNAs targeted circRNA8077 (Fig. 6b).

### Validation of circRNA with RNase R and RT-qPCR

**Fig. 7:**
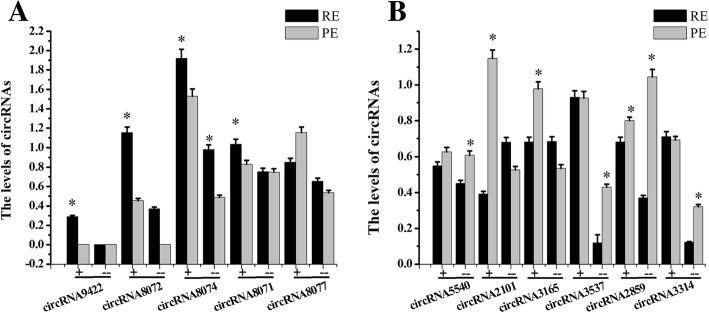
Validation of circRNA with RNase R and RT-qPCR. Note: “+” suggested that the total RNA were incubated for 15 min at 37 °C with 3 U/μg of RNase R; “--” mean the RNA did not treat with RNase R; “*” suggested *P <* 0.05 between RE and PE. GAPDH was used as internal control gene for normalization in this experiment. Values were “means ± SD” in receptive endometrium that were relative to pre-receptive endometrium. (**a**) showed the levels of circRNA9422, circRNA8072, circRNA8074, circRNA8071, circRNA8077; (**b**) showed the levels of circRNA5540, circRNA2101, circRNA3165, circRNA3537, circRNA2859, circRNA3314

To verify whether the back-spliced events were indicative of true circular molecules, we examined the physical properties of these products using two independent methods. First, circRNAs are known to be strongly resistant to the exonuclease RNase R compared with linear RNAs. The circRNA candidates in this study were also resistant to RNase R treatment and the abundance of linear RNAs decreased, thus confirming the circularized characteristics of these molecules. Second, because the circRNAs have a special head-to-tail junction site that differs from canonical linear splicing transcripts, RT-qPCR was used to validate circRNA candidates via the targeted amplification of the head-to-tail junction region unique to circRNA. To this end, outward-facing primers (Additional file 11) were designed against 11 circRNA candidates with high expression levels, followed by standard Sanger sequencing. Combining the two approaches, the results showed that the eleven circRNAs were consistent with the rRNA-depleted sequencing data, except circRNA8077 (Fig. 7).

### Correlation analysis of circRNAs and their hosting genes

**Fig. 8:**
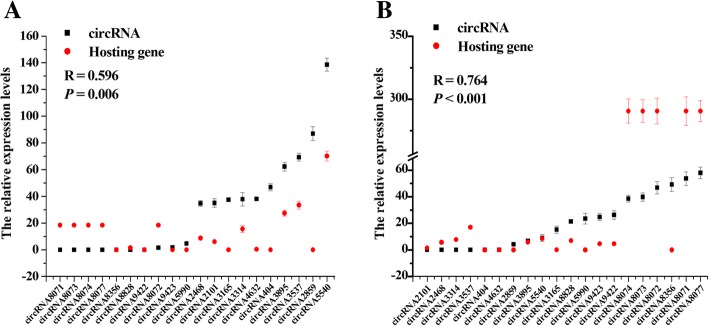
The correlation analysis of circRNAs and their hosting genes in the endometrium tissue of dairy goats. Note: The expression levels of circRNAs and their hosting genes were measured in each of 3 dairy goats in pre-receptive (PE) and receptive (RE) endometrium. Pearson analysis using the SPSS software was performed to identify the correlation coefficient. (**a**) PE, (**b**) RE

To validate the global downregulation of circRNAs in goat endometrial tissues, we selected the upregulated circRNAs with the ten highest FPKM values in the RE and the downregulated circRNAs with the ten highest FPKM values in the PE. To better evaluate the relationship between the circRNAs and their hosting genes, data on mRNA expression in the endometrial tissue from goats at PE and RE phases were used for the correlation analysis. A strong correlation was observed between the circRNAs and their hosting genes in the PE (R = 0.596, *P* = 0.006, Fig. 8a) and RE (R = 0.764, *P <* 0.001, Fig. 8b). These results indicated that circRNAs and their host genes may be co-regulated in the goat endometrium.

### The effect of E2 and P4 on the expression levels of circRNAs in goat EECs and ESCs

**Fig. 9:**
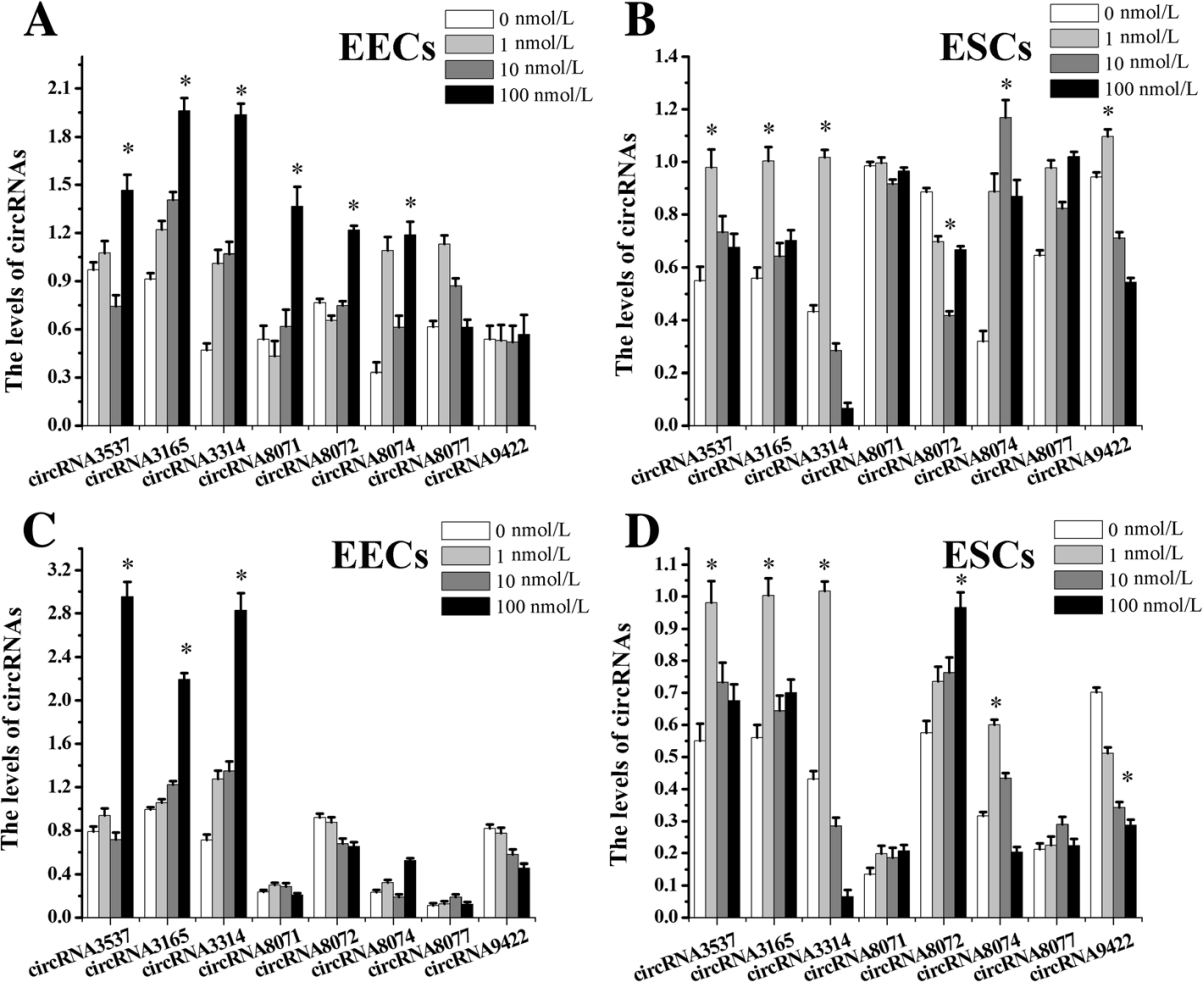
The effect of E2 and P4 on the expression levels of circRNAs in EECs and ESCs. Note: **a** mean the EECs were treated with different concentrations of estradiol (E2); **b** mean the ESCs were treated with different concentrations of E2. **c** mean the EECs were treated with different concentrations of progesterone (P4); **d** mean the ESCs were treated with different concentrations of P4. “*” suggested *P <* 0.05 between the different concentrations of E2/P4, and was marked on the highest value. GAPDH was used as internal control gene for normalization in this experiment. Values were “means ± SD” in receptive endometrium that were relative to pre-receptive endometrium

The endometrium is composed mainly of two types of cells, ESCs and EECs, and these cells are regulated by E2 and P4. In EECs, the levels of circRNA3537, circRNA3165, circRNA3314, circRNA8071, circRNA8072, and circRNA8074 were up-regulated by 100 nmol/L E2, while circRNA8077 and circRNA9422 levels were not changed (Fig. 9a). In ESCs, the levels of circRNA3537, circRNA3165, circRNA3314, and circRNA9422 were up-regulated by 1 nmol/L E2, circRNA8072 was down-regulated and circRNA8074 was up-regulated by 10 nmol/L E2, and circRNA8071 and circRNA8077 levels were not changed (Fig. 9b). In EECs, the levels of circRNA3537, circRNA3165, and circRNA3314 were up-regulated by 100 nmol/L P4 (Fig. 9c). The levels of circRNA3537, circRNA3165, circRNA3314 and circRNA8074 were also up-regulated by 1 nmol/L P4 in ESCs. After 100 nmol/L P4 treatment, circRNA8072 levels were up-regulated, circRNA9422 levels were downregulated, and circRNA8071 and circRNA8077 levels were not changed (Fig. 9d).

## Discussion

This study yielded 18 Gb sequence for every sample using RNA-Seq, representing approximately seven times the size of the goat genome. After separating out and discarding low-quality sequences, we obtained ~ 90 million clean reads, and the sequencing depth in this study was sufficient to detect the transcripts expressed at low levels [45].

A recent study reported 65,731 human circRNAs in brain tissues [20], and another study further detected at least 27,296 circRNA candidates in six normal tissues and seven cancer tissues [46]. Compared with human studies, fewer circRNAs (21,813 distinct circRNA candidates) were identified in goat. This could be due to the fact that the human genome is better annotated than the goat genome. Moreover, the human circRNAs were derived from various tissues [46], but the circRNAs in the present study were only obtained from two physiological stages of the goat endometrium. In addition, the expression of circRNAs was developmentally regulated and varied between tissues [22, 47, 48]. Notably, 5,925 circRNAs were specifically identified in the RE, and 9,078 were specifically identified in the PE, but only 6,810 circRNAs were co-expressed at both stages, exhibiting developmental stage-specific expression consistent with previous studies [49, 50]. This indicates that these molecules may play crucial roles in goat endometrium development.

Because a circRNA comes from a given linear transcript, it is likely that the circRNA function is associated with its hosting gene function [22]. *CRIM1*, the hosting gene of circRNA8077, plays an essential role in activating the expression of vascular endothelial growth factor and ECM [51], which are important during early pregnancy [52, 53]. *CRIM1* was also the hosting gene of circRNA8072, a markedly upregulated circRNA with a high FPKM value. Importantly, we found that half of the upregulated circRNAs with the ten highest FPKM values in the RE were derived from CRIM1. This indicated that multiple circularized exons could be produced from a single gene locus, which is consistent with previous literature [54, 55]. Collectively, these results suggested that *CRIM1* might play an important regulatory role in the endometrial receptivity of dairy goats by generating alternative circularization products and extending the complexity of post-transcriptional regulation. However, further study is required as the present study provides no direct evidence that *CRIM1* and its circRNAs participated in the formation of the RE.

The downregulated circRNA with the highest FPKM value in the PE was circRNA5540, showing 15.37-fold decreased expression in the RE. Its hosting gene, *OGDH*, was one of the top ten transcripts with the highest expression values in the bovine endometrium [56]. This gene determines the metabolically active endometrial phenotype, which plays an important role in the formation of RE [56]. In addition, a previous study showed that *OGDH* mRNA expression increased 2.15-fold [26] in the RE compared to the PE. Therefore, based on both the previous and present data, we propose that *OGDH* contributes to the formation of endometrial receptivity by decreasing its noncoding transcript, circRNA5540, thereby increasing its mRNA level. However, this hypothesis should be validated under well-controlled conditions in animal models.

Gene Ontology (GO) provides a simple and quick way to understand the properties of genes and gene products, and it is widely used as a classification system for gene function [57]. Extracellular matrix structural constituent (GO:0005201) and integrin-mediated signaling pathway (GO:0007229) came from the GO analysis of hg-DECs in this study. These pathways interested us because integrins have been implicated as a biomarker of RE and participate in the remodeling of the ECM in the endometrium during early pregnancy [26]. A previous study reported that a number of genes that encoded ECM proteins were enriched in the bovine endometrium during the estrous cycle [58]. Importantly, all of these endometrial events were mediated by mechanisms of proliferation, differentiation, and apoptosis. *PTEN* (phosphatase and tensin homolog deleted on chromosome ten) antagonized *PI3K* (phosphatidylinositol 3-kinase) activity and negatively regulated serine/threonine kinase *Akt* [59], which modulated the activity of a variety of downstream proteins that were related to cell survival and proliferation [60]. Therefore, the inclusion of the annotation for phosphatidylinositol-3,4,5-trisphosphate binding (GO:0005547) was also interesting to determine the important roles of the PI3K/Akt signaling pathway in the endometrium.

KEGG pathway analysis is widely used to understand the interaction between a set of genes in the context of biological functions [38, 61]. The KEGG pathway analysis of hg-DECs in this study revealed that several genes might be involved in several cancer-related pathways and regulate the development of the endometrium though their cognate circRNAs. There were striking similarities between the behavior of placental cells during the “window of implantation” and that of cancer cells diffusion [62]. An appreciation of the cancer mechanisms may lead to a better understanding of the maternal mechanisms that control embryo implantation. Moreover, a previous study proposed that the cellular mechanisms used by trophoblast cells during implantation are reused by cancer cells to invade and spread within the body [63]. Integrins, matrix metalloproteinase, ECM, and angiogenesis are common features of both implantation and the spread of cancer [63, 64].

Recent studies have confirmed that some circRNAs exert their biological effects by competitively binding miRNA, thus releasing and increasing the expression levels of the miRNA target genes [65–67]. This miRNA sponge mechanism has been clearly demonstrated for circRNAs ciRS-7/CDR1as and circRNA Sry [16, 28]. In the present study, 99.41% (21,683) of the identified circRNAs were predicted as putative targets for 436 goat miRNAs, suggesting that a miRNA was regulated by more than one circRNA [46], and further analysis showed that a circRNA could serve as a sponge for multiple miRNAs. However, further research is needed as effective miRNA sponging by exonic circRNAs may be relatively unusual or may not require a large number of miRNA binding sites [17]. Moreover, we observed that 308 DECs were predicted as targets of 141 differentially expressed miRNAs between the RE and PE [15], suggesting that these molecules play important roles in the formation of endometrial receptivity. Moreover, 16 DECs were predicted to be regulatory factors of miR-449a that were significantly upregulated in the RE [15]. Surprisingly, circRNA8072, circRNA8073, and circRNA8074 were significantly upregulated in the RE and were also predicted to be targets of miR-449a. Further study showed that circRNA8073 could bind miR-449a via the target site and inhibit miR-449a activity, creating a negative feedback relationship between circRNA8073 and miR-449a in EECs. Furthermore, circRNA8073 could increase the expression levels of centrosomal protein55 (*CEP55*) by sponging miR-449a in EECs *in vitro*. Moreover, circ-8073/miR-449a/*CEP55* could regulate EECs proliferation and apoptosis via the PI3K/AKT/mTOR pathway. Taken together, circRNA8073 could regulate *CEP55* by sponging miR-449a to promote EEC proliferation via the PI3K/AKT/mTOR pathway [68].

In addition, circRNAs substantially contributed to total RNA [69] and circular isoforms typically accounted for 5–10% of the total transcripts of their corresponding coding genes. However, certain circRNAs were up to 200 times more abundant than their linear counterparts [47]. In this study, we found no distinct difference in the expression level of circRNAs and their hosting gene mRNA in the PE, but the expression levels of the hosting genes were higher than the levels of circRNAs in the RE. We suspect that this occurs because circRNAs comprise one to several coding exons of linear mRNAs and range between a few hundred and thousands of nucleotides in length [21]. However, circRNAs aligned with antisense, intronic, or intergenic sequences, or untranslated regions have also been described. Nevertheless, circular RNA transcripts were generally expressed at low levels compared with linear RNAs [47], and our results support this observation.

CircRNAs are RNA molecules with covalently joined 3′ and 5′ ends formed by back-splice events, thus presenting as covalently closed continuous loops [69]. Both mechanisms of “exon skipping” and “direct back-splicing” involve back-splicing by the canonical spliceosome [27], and a majority of apparent back-splice sequences in RNA-Seq data derive from circRNAs [17]. We validated ten of eleven (91%) circRNA candidates by the targeted amplification of the head-to-tail junction region unique to the circRNAs using reverse transcription-PCR with an outward-facing primer and standard Sanger sequencing. Furthermore, some circRNAs were regulated by E2/P4 in EECs and ESCs, and each circRNA molecule exhibited unique regulation characteristics with respect to E2 and P4 *in vitro*.

## Conclusion

In this study, high-quality circRNA expression profiles were obtained from the endometrial tissues of dairy goats. We identified 21,813 circRNAs, 334 of which were differentially expressed between the PE and RE. GO and KEGG analyses, the interaction network analysis of circRNA-miRNA, and the correlation analysis of circRNAs and their hosting genes could contribute to a better understanding of how circRNAs mediate the regulation of target genes in the development of endometrium receptivity. Additional studies showed that some circRNAs were regulated by E2/P4 in endometrial cells. Taken together, these findings reveal a new level of diversity in the goat transcriptome and introduce new insights into their regulation during the development of endometrial receptivity.

## Additional files

Additional file 1: **Table S1.** The groups of hormone treatment (nmol/L) (DOCX 15 kb)

Additional file 2: **Figure S1.** The overview of the results of RNA-Seq. (A) Principal component analysis (PCA) of the results of RNA-Seq. The six independent endometrium samples collected from 3 goats at gestational day 5 (PE, GE258-C, GE796-C, GE990-C) and 3 goats at gestational day 15 (RE, GE484-T, GE706-T, GE5928-T), respectively. (B) Boxplot of the results of RNA-Seq for four endometrium samples. Boxplot of the log_10_ FPKM (Fragments Per Kilobase of exon per million fragments mapped) expression values in four endometrium samples. The Fig. reflects the distribution of FPKM values computed for the circRNAs in each samples from RNA-Seq data, and shows that the median of the expression values across the samples being compared for differential expression are comparable. (TIF 2658 kb)

Additional file 3: **Table S2.** The result of nonlinear alignment using TopHat-Fusion. (XLS) (XLS 24 kb)

Additional file 4: **Table S3.** CircRNA expression in goat endometrium. (XLS) (XLS 9770 kb)

Additional file 5: **Table S4.** Differential expressed circRNA between receptive and pre-receptive endometrium. (XLS) (XLS 1658 kb)

Additional file 6: **Table S5.** CircRNA specifically expressed in the RE or PE. (XLS) (XLS 3082 kb)

Additional file 7: **Table S6.** Enrichment analysis of hgDEC. (XLS) (XLS 420 kb)

Additional file 8: **Table S7.** Sgnificant GO terms of hgDEC. (XLS) (XLS 177 kb)

Additional file 9: **Table S8.** KEGG significant analysis of hgDEC. (XLS) (XLS 58 kb)

Additional file 10: **Table S9.** All predicted miRNAs bind to circRNAs in goat. (XLS) (XLS 9205 kb)

Additional file 11: **Table S10.** The primers used in this study. (XLS) (XLS 26 kb)

## Abbreviations

ALDH18A1: Aldehyde dehydrogenase 18 family member A1
circRNAs: circular RNAs
CRIM1: Cysteine-rich motor neuron 1
DAG: Directed acyclic graph
DECs: Differentially expressed circRNAs
E2: Estrogen
ECM: Extracellular matrix
EECs: Endometrial epithelium cell lines
ESCs: Endometrial stromal cell line
FPKM: Fragment Per Kilobase of exon per Million fragments mapped
hgDECs: Hosting genes of differentially expressed circRNAs
OGDH: 2-oxoglutarate dehydrogenase
P4: Progesterone
PE: Pre-receptive endometrium
PI3K: Phosphatidylinositol 3-kinase
PTEN: Phosphatase and tensin homolog deleted on chromosome ten
RE: Receptive endometrium
