# Supplementary material for: Analyses of circRNA profiling during the development from pre-receptive to receptive phases in the goat endometrium
Source: J Anim Sci Biotechnol. 2019 Apr 25;10:34. doi: 10.1186/s40104-019-0339-4 (PMC6482587; doi:10.1186/s40104-019-0339-4)
Supplement: Supplementary file 1 — Table S1. The groups of hormone treatment (nmol/L) (DOCX 15 kb) [file 40104_2019_339_MOESM1_ESM.docx]

**Additional file 1: Table S1**

**Additional Table 1 | The groups of hormone treatment (nmol/L)**

| **Groups** | **1** | **2** | **3** | **4** |
| --- | --- | --- | --- | --- |
| **estrogen (E2)** | 0 | 1 | 10 | 100 |
| **Progesterone** **(P4)** | 0 | 1 | 10 | 100 |
