# Supplementary figures and images for: Analyses of circRNA profiling during the development from pre-receptive to receptive phases in the goat endometrium
Source: J Anim Sci Biotechnol. 2019 Apr 25;10:34. doi: 10.1186/s40104-019-0339-4 (PMC6482587; doi:10.1186/s40104-019-0339-4)

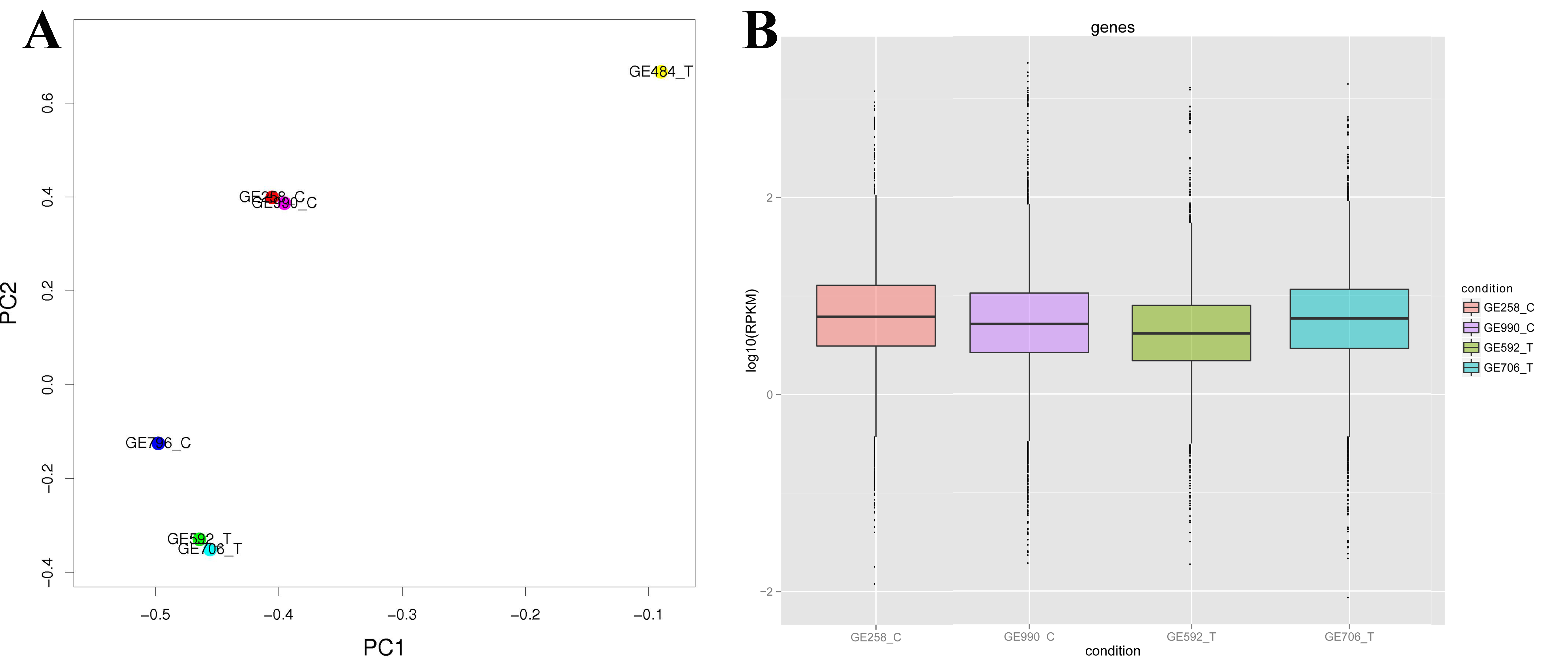

Supplement: Supplementary file 2 — Figure S1. The overview of the results of RNA-Seq. (A) Principal component analysis (PCA) of the results of RNA-Seq. The six independent endometrium samples collected from 3 goats at gestational day 5 (PE, GE258-C, GE796-C, GE990-C) and 3 goats at gestational day 15 (RE, GE484-T, GE706-T, GE5928-T), respectively. (B) Boxplot of the results of RNA-Seq for four endometrium samples. Boxplot of the log10 FPKM (Fragments Per Kilobase of exon per million fragments mapped) expression values in four endometrium samples. The Fig. reflects the distribution of FPKM values computed for the circRNAs in each samples from RNA-Seq data, and shows that the median of the expression values across the samples being compared for differential expression are comparable. (TIF 2658 kb) [file 40104_2019_339_MOESM2_ESM.tif]
